# Sepsis outbreak following a probable extrinsic contamination of propofol by *Escherichia coli*, Geneva, 2024

**DOI:** 10.1186/s13756-025-01655-x

**Published:** 2025-12-24

**Authors:** Roberta Petrucci, Amir Allaoua, Aliki Metsini, Abdessalam Cherkaoui, Diem-Lan Vu, Vladimir Lazarevic, Delphine Perreard, Nechan Haroutunian, Sophie Garcin, Olivier Grosgurin, Nathalie Vernaz, Stephan Harbarth, Simon Regard, Jacques Schrenzel, Alessandro Cassini

**Affiliations:** 1 Cantonal Physician’s Office, Cantonal Health Department, Geneva, Switzerland; 2 Division of Emergency Medicine, Department of Acute Care Medicine, Geneva University Hospitals, Geneva, Switzerland; 3 Bacteriology Laboratory, Division of Laboratory Medicine, Department of Diagnostics, Geneva University Hospitals, Geneva, Switzerland; 4 Paediatric Infectious Diseases Unit, Children’s Hospital, Geneva University Hospitals, Geneva, Switzerland; 5 Genomic Research Laboratory, Department of Medicine, Geneva University Hospitals and Faculty of Medicine, Geneva, Switzerland; 6 Infection Control Program, Geneva University Hospitals and Faculty of Medicine, Geneva, Switzerland; 7 Cantonal Pharmacist’s Office, Cantonal Health Department, Geneva, Switzerland; 8 Division of Clinical Pharmacology and Toxicology, Geneva University Hospitals, Geneva, Switzerland

## Abstract

Over a 14-day period, four patients developed sepsis within hours of undergoing endoscopic procedures at a gastroenterology outpatient clinic in Geneva (Switzerland), triggering an urgent epidemiological investigation upon notification to authorities. The case clustering raised concerns about a iatrogenic source, prompting a coordinated public health response. This report outlines the investigation led by the Geneva Health Authorities in collaboration with the medical team, emergency care providers, infection prevention specialists, and the bacteriology laboratory at Geneva University Hospitals. A multidisciplinary team, including an epidemiologist, a pharmacist and an infection prevention specialist, conducted an on-site investigation the day after the outbreak was identified, highlighting a probable extrinsic contamination of propofol by *Escherichia coli.*

**Supplementary Information:**

The online version contains supplementary material available at 10.1186/s13756-025-01655-x.

## Cluster description

Following the identification of the epidemiological link between two cases of sepsis by emergency physicians, two additional cases were found through retrospective reviews of hospital records, patient interviews, and consultations with the clinic's medical team. All cases were classified as sepsis occurring within 24 h post-endoscopy. Sepsis was defined as life-threatening organ dysfunction caused by a dysregulated host response to infection, in accordance with the Sepsis-3 criteria [1].

The four cases occurred on three different days over a two-week period (days 1, 12, and 13, with two cases on the last day). Among 28 patients who underwent an endoscopic procedure under propofol at the same clinic during the three days under investigation, four developed sepsis within 24 h post-procedure. None of the remaining 24 patients, contacted by telephone, reported any symptoms. Days without new cases during the two-week period between events were not investigated, as the standard 24–48-h post-procedure follow-up calls reported no symptomatic patients. The same team (one doctor and two nurses, alternating roles in assistance) performed all the endoscopies.

The four cases included three women and one man, aged 40 to 59 years old. Three developed symptoms at the clinic during the 30-min post-procedure observation period, while one experienced symptoms shortly after returning home. Symptoms ranged from fever and chills to nausea, abdominal pain, hematemesis, and diarrhoea. Two patients were admitted the next day, while two were admitted on the day of the procedure. All were diagnosed with sepsis, with varying degrees of acute kidney failure and severe inflammatory syndrome. No organ-specific infectious focus was identified in any patient. All patients survived and were discharged between 7 and 15 days after hospitalisation. (Table 1, supplementary material).

## Epidemiological investigation

The investigation was led by a public health specialist and carried out by an interdisciplinary team. It began the day following the identification of the last case and assessed endoscope reprocessing protocols, medication preparation and storage practices, staff training and compliance with aseptic techniques, and clinic infrastructure, including the separation of clean and contaminated areas.

Observed breaches in infection prevention and control practices included the absence of standard operating procedures; deviation from the manufacturer’s and IPC recommendations (such as prefilling syringes twice daily instead of immediately before each procedure, lack of labelling or documentation of preparation time, and no record of the operator preparing the medication); deficiencies in staff training (with missing or expired aseptic technique certification); and stock management failures (including reports of sterile supply shortages, sterile syringe caps unavailable during inspection, and prefilled syringes stored at room temperature).

Given the clinical presentation and the timing of symptom onset, the most probable cause of this sepsis outbreak was bacterial contamination of an intravenously administered therapeutic product. Three potential sources were identified: propofol, saline solution, and hyoscine butylbromide. While the first two products were administered to all patients undergoing an endoscopy procedure, the latter was not administered to all affected patients and was therefore ruled out. Saline solution is a less favourable medium for bacterial growth, making propofol the most likely source of contamination.

Other hypotheses initially formulated (sepsis due to bacterial contamination of endoscopic equipment and toxic shock due to direct chemical contamination of endoscopic equipment) were not supported by the investigation and were discarded.

## Microbiological analysis

Blood cultures (BC) were performed on samples collected from the four affected patients during hospitalisation (Table 1, supplementary material). In addition, BC bottles (BD BACTEC™ Plus Aerobic medium) were inoculated with 5ml of the syringes and sealed ampoules collected on-site on the day of the investigation, to assess the potential source of contamination. Inoculated bottles were immediately placed in the BACTEC FX instrument (Becton, Dickinson and Company) for 14 days. For *Escherichia coli*, BC bottles turned positive after 2h incubation in the instrument. Blood culture fluid was aspirated from each positive bottle with sterile needle/syringe and transferred into a sterile tube in order to be inoculated on solid culture media using the WASPLab. No open saline solutions were available for analysis. Among the three patients with negative blood cultures, two received antibiotics 7 and 1.5 h before the first sampling, respectively, while one received antibiotics afterward. One patient's blood culture grew *Escherichia coli*.

Out of the 8 syringes analysed, 3 tested positive for *E. coli*, one for *Micrococcus luteus*, one for *Streptococcus parasanguinis*, and 3 tested negative. All 5 sealed ampoules tested negative.

Following whole-genome sequencing, core genome single nucleotide polymorphisms (cgSNP) analysis revealed no single nucleotide variants (SNVs) between the *E*. *coli* isolates from a patient’s blood culture and the three contaminated propofol syringes, consistent with clonal origin. Next-generation sequencing-based genotyping indicated that the isolate belonged to MLST type 1155, serotype O39:H4, and FimH type 20, with no virulence factors characteristic of EHEC, EPEC, EIEC, ETEC, or EAEC detected.

## Discussion

This outbreak underscores the risk of propofol-associated infections due to suboptimal preparation and administration procedures. While definitive conclusions cannot be drawn, genetic analysis confirmed a common *E. coli* strain in both contaminated syringes and a patient’s bloodstream, strongly suggesting that the direct injection of bacteria during propofol administration was the most probable cause. The rapid onset of symptoms following the procedure further supports this as the source of infection. The fact that not all patients who received the propofol solution became contaminated strengthens the hypothesis that the contamination occurred during the final preparation of the syringes, due to inadequate IPC practices, rather than originating from the solution itself. Contamination most likely resulted from faecal contamination due to poor hand hygiene. A less probable cause was drug preparation in areas also used for endoscope reprocessing.

Propofol has long been recognized as a significant risk factor for healthcare-related infections following various procedures [2–4]. Between 1989 and 2014, 20 outbreaks were reported worldwide, affecting 144 patients and resulting in 10 deaths, though many cases likely remain unreported. Infections have been linked primarily to endoscopic procedures. Most data come from operating rooms and intensive care units [5], with limited outpatient reports, likely due to reporting bias.

Propofol’s lipid emulsion provides an ideal medium for bacterial growth [6]. Reported infections have involved multiple pathogens, including bacteria (47.2%), Candida albicans (21.5%), and HCV/HBV (22.3%), occasionally co-occurring in the same outbreak. In many cases, microbiological confirmation was lacking, possibly due to prompt antimicrobial treatment or delayed blood cultures [5].

Microbiological contamination of propofol can occur either during manufacturing (intrinsic) or after vial opening (extrinsic), with the latter being more common. Studies have shown a significant increase in risk when manufacturer guidelines are not followed [3, 5].

Several risk factors have been identified, including prolonged storage of prepared syringes, inadequate sterile technique during drug preparation, reuse of syringes or infusion lines, and contamination of infusion ports or multi-dose vials. Delayed administration can increase bacterial contamination levels by 20–26% after 12 h [7].

In the present outbreak, epidemiological patterns suggest extrinsic rather than intrinsic contamination, as sealed ampoules tested negative. The presence of the same *E. coli* strain in contaminated syringes and a patient’s blood culture strengthens the causal link between propofol administration and the four episodes of sepsis. The absence of bacterial identification in the other patients may be due to prior antibiotic treatment, as blood culture positivity drops from 24.3% before antibiotics to 9.1% within five hours [8]. In addition to the three syringes contaminated with *E*. *coli*, microbiological analysis also detected *M*. *luteus* and *S*. *parasanguinis*—commonly found on the skin and in the oral/respiratory tract, respectively—in two other syringes, highlighting concerns about inadequate aseptic procedures.

The cause of contamination was likely multifactorial, with several deficiencies identified, including a lack of standardized handling protocols and certified nurse training, poor adherence to aseptic procedures, and the preparation of multiple syringes at once. A shortage of sterile material (e.g., syringe caps) was observed on the day of the investigation. While this may have contributed to contamination during the procedures, verification was not possible, as stock shortages were not documented.

This study has several limitations. First, case identification relied on passive case finding and a non-enhanced review of potentially exposed patients, which may have led to under-ascertainment. Second, data on storage conditions and culturing of the injectable products (propofol and saline) were limited. Finally, only one of the four patients had microbiological confirmation linking their infection to the outbreak, which restricts the strength of causal inference.

This outbreak highlights the risks associated with suboptimal aseptic practices in outpatient endoscopy settings. Implementing strict aseptic handling protocols can reduce contamination risks [9]. The following measures were implemented: immediate suspension of endoscopic procedures; sharing audit reports with the involved medical team to guide them in evaluating and improving hygiene practices, stock management, drug administration, and staff training to ensure compliance with standards. Following notification to the local gastroenterology association, a communication was sent to all affiliates to raise awareness and reinforce adherence to national quality and hygiene standards.

The outbreak was swiftly managed by a multidisciplinary team of epidemiologists, quality inspectors, pharmacists, and medical device experts, in collaboration with the clinic's medical team. Recommendations were promptly communicated, enabling the implementation of corrective measures without delay. Collaboration with the professional association turned the incident into an opportunity to promote best practices among outpatient endoscopy professionals and improve patient safety.

## Supplementary Information

Below is the link to the electronic supplementary material.

Supplementary Material 1

## Data Availability

No datasets were generated or analysed during the current study.
