# Supplementary material for: Sepsis outbreak following a probable extrinsic contamination of propofol by Escherichia coli, Geneva, 2024
Source: Antimicrob Resist Infect Control. 2025 Dec 24;14:151. doi: 10.1186/s13756-025-01655-x (PMC12729812; doi:10.1186/s13756-025-01655-x)
Supplement: Supplementary file 1 — Supplementary Material 1 [file 13756_2025_1655_MOESM1_ESM.docx]

Supplementary Table 1. Chronology of events: procedures, symptom onset, bacteriological sampling and analysis, and antibiotic treatment.

| **Case** | **Procedure**  **Date*, time** | **Beginning of symptoms**  **Day, time** | **Hospitalisation**  **Day, time** | **Blood culture**  **Day, time**  **Result** | **Antibiotic treatment**  **Day, time** | **Clinical findings** |
| --- | --- | --- | --- | --- | --- | --- |
| 1 | Gastroscopy  Day 1, 8am | Minutes after the end of the procedure, in the observation room | Day 2, 7.04pm | Day 2, 7.24pm (IV line)  Day 3, 7.02pm (central catheter)  Day 3, 7.02pm (arterial catheter)  All sterile | Day 2, 8.23 pm  Ceftriaxone  Day 2, 9.15 pm  Piperacillin/Tazobactam | Chills, vomiting, abdominal pain, fever.  No clinical sign of infectious focus with negative thoraco-abdomino-pelvic CT. |
| 2 | Colonoscopy  Day 12, 8am | Few hours after the end of the procedure, once at home | Day 13, 8.56am | Day 13, 6.50pm  Sterile | Day 13, 11.44 am  Piperacillin/Tazobactam | Fatigue, chills, repeated vomiting, hypotension.  Lactic acidosis, inflammatory syndrome.  No clinical sign of infectious focus with negative thoraco-abdomino-pelvic CT. |
| 3 | Gastroscopy  Day 12, 1.30pm | Minutes after the end of the procedure, in the observation room | Day 13, 4.43pm | Day 13, 7.40pm  Sterile | Day 13, 6.07 pm  Co-amoxiclav  Day 13, 7.31 pm  Amikacin/Vancomycin  Day 13, 9.43 pm Piperacillin/Tazobactam | Fatigue, chills, abdominal pain, profuse diarrhoea, hypotension.  Lactic acidosis, inflammatory syndrome, liver cytolysis.  No clinical sign of infectious focus with negative thoraco-abdomino-pelvic CT. |
| 4 | Colonoscopy  Gastroscopy  Day 13, 1.30pm | Minutes after the end of the procedure, in the observation room | Day 13, 5.35pm | Day 13, 8.05 pm  Positive for *Escherichia coli*  Day 14, 4.25 pm  Sterile | Day 13, 6.49 pm  Amikacin/Co-amoxiclav  Day 13, 7.34 pm  Vancomycin  Day 14. 00.05 am Piperacillin/Tazobactam | Fever, hypotension, chills, profuse diarrhoea, vomiting.  Lactic acidosis, inflammatory syndrome.  No clinical sign of infectious focus with negative thoraco-abdomino-pelvic CT. |

* In this report, day numbers are referenced relative to the outbreak day.
